# Highly emissive excitons with reduced exchange energy in thermally activated delayed fluorescent molecules

**DOI:** 10.1038/s41467-019-08495-5

**Published:** 2019-02-05

**Authors:** Anton Pershin, David Hall, Vincent Lemaur, Juan-Carlos Sancho-Garcia, Luca Muccioli, Eli Zysman-Colman, David Beljonne, Yoann Olivier

**Affiliations:** 1 Laboratory for Chemistry of Novel Materials, University of Mons, Place du Parc 20, B-7000 Mons, Belgium; 2 Organic Semiconductor Centre, EaStCHEM School of Chemistry, University of St Andrews, St Andrews, KY16 9ST United Kingdom; 3 Departamento de Química Física, Universidad de Alicante, E-03080 Alicante, Spain; 4 Dipartimento di Chimica Industriale “Toso Montanari”, Università di Bologna, I-40136 Bologna, Italy

## Abstract

Unlike conventional thermally activated delayed fluorescence chromophores, boron-centered azatriangulene-like molecules combine a small excited-state singlet-triplet energy gap with high oscillator strengths and minor reorganization energies. Here, using highly correlated quantum-chemical calculations, we report this is driven by short-range reorganization of the electron density taking place upon electronic excitation of these multi-resonant structures. Based on this finding, we design a series of π-extended boron- and nitrogen-doped nanographenes as promising candidates for efficient thermally activated delayed fluorescence emitters with concomitantly decreased singlet-triplet energy gaps, improved oscillator strengths and core rigidity compared to previously reported structures, permitting both emission color purity and tunability across the visible spectrum.

## Abstract (web summary)

Thermally activated delayed fluorescence is a mechanism for enhancing the efficiency of organic light emitting diodes by harvesting triplet excitons, but there is still a need to design more efficient materials. Here, the authors rationally design and characterize a series of π-extended boron- and nitrogen-doped nanographenes as promising candidates.

## Introduction

The discovery of purely organic thermally activated delayed fluorescence (TADF) materials, with its premise to break the spin statistical bottleneck of 25% internal quantum efficiency without the requirement of rare noble metal emitters, has prompted a paradigm shift in the design of emitter materials for use in organic light-emitting diodes (OLEDs)^1^. TADF is rooted in a thermally promoted reverse intersystem crossing (RISC) process enabling upconversion of triplet excitons into emissive singlet excitons, which otherwise would be lost through non-radiative pathways. One key property TADF molecules should fulfill for efficient RISC is a small energy gap between the lowest singlet and triplet excited states Δ*E*_ST_ (usually < 0.2 eV), so that delayed fluorescence can be thermally activated at room temperature (though exceptions to this general rule exist that involve higher-lying triplet states)^2^. The most widely applied design strategy so far is based on molecules featuring weakly coupled and spatially separated donor (D) and acceptor (A) moieties. This motif sustains charge-transfer (CT) excitations with small exchange interactions and correspondingly small Δ*E*_ST_ compared to localized excited states^3–5^.

However, this approach suffers from a number of drawbacks that directly reflect the nature of the emissive excited state. In particular, achieving high photoluminescence quantum yields (PLQY) and color purity in conventional TADF molecule-based OLEDs has been challenging thus far^6–9^. As a consequence of their dominant CT character, the singlet electronic excitations in these molecules often display fairly small radiative emission cross-sections (oscillator strengths, *f*_osc_). Hence, high PLQY in these materials is only possible through efficient suppression of non-radiative decay processes. Moreover, charge-transfer excitations are usually accompanied by large structural reorganization, associated with conformational degrees of freedom in D-A molecular architectures, leading to broad emission spectra. Yet, such conformational gating effects also feed small admixtures of local (covalent) excited-state character into (primarily triplet) CT (ionic) excitations, prompting the needed spin-orbit interaction that mediates spin conversion^10–13^. Thus, designing optimally performing TADF compounds in this scenario relies on the necessarily delicate balance between antagonistic effects.

**Fig. 1:**
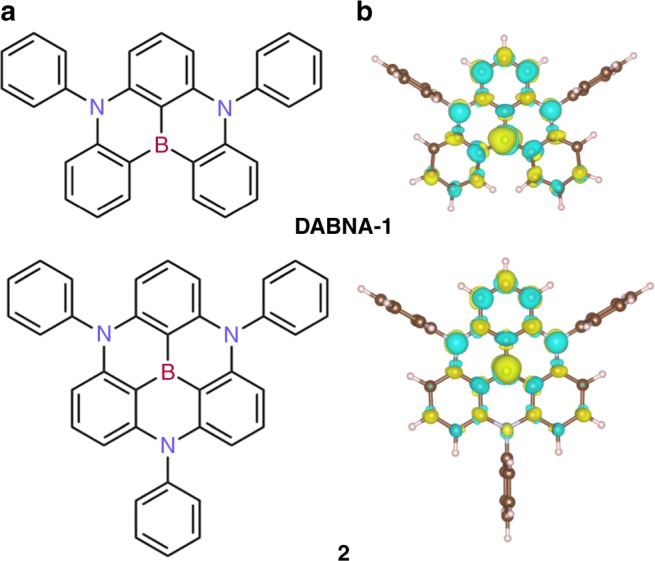
Evidence for short-range charge transfer in boron-centered azatriangulene molecules. **a** Chemical structures and **b** difference density plots of **DABNA-1** and **2** (summing over the two doubly degenerated S_1_ states, see Supplementary Fig. 4). Yellow (blue) color indicates increased (decreased) electron density upon S_0_-S_1_ excitation

A notable departure from the usual strategy has recently been proposed by Hatakeyama et al.^6,7,14^ who designed TADF emitters as triangulene cores incorporating *ortho*-substituted boron and nitrogen atoms to promote multiple resonance effects (see chemical structures in Fig. 1 and resonant structures in Supplementary Fig. 5). Unlike conventional D-A architectures, these boron-centered azatriangulenes yield concomitantly narrow emission spectra (full-width at half-maximum = 28 nm), relatively large *f*_osc_ (~0.2 for both **DABNA-1** and **2**) and small Δ*E*_ST_ (~0.15 eV for **DABNA-1** and 0.21 for **2**) values. OLEDs with high maximum external quantum efficiencies (EQE_max_) of 13.5% and pure blue emission [CIE coordinates of (0.13, 0.09] for **DABNA-1** were demonstrated. In marked contrast with experiment, recent publications^5,15,16^ reported Time-Dependent Density Functional Theory (TD-DFT) Δ*E*_ST_ predictions in the range 0.4–0.6 eV depending on functional, largely overestimating the experimental values. Based on these TD-DFT calculations, two mechanistic pictures for TADF in boron-centered azatriangulenes molecules have been put forward: Lin et al.^15^ claimed that RISC occurs directly from T_1_ to S_1_ while Northey et al. advanced that RISC involves an intermediate higher-lying triplet state T_2_^16^.

Here, by scrutinizing the nature of the lowest electronic excitations in these molecules, we propose a new paradigm for TADF that builds on the concept of short-range charge-transfer illustrated in Fig. 1. While long-distance charge separation in multichromophoric D-A systems reduces Δ*E*_ST_ at the expense of *f*_osc_, we show that boron-centered azatriangulenes undergo a large but local spatial reorganization of the electronic density in the excited states, which significantly lowers the exchange energy while maintaining high overlap between wavefunction tails and therefore high *f*_osc_ transitions, together with small structural relaxation energies. Based on these results, we propose analogs that we hope will lead to high performance TADF emitters in OLED devices.

## Results

### Origin of small Δ*E*_ST_ in DABNA molecules

We start our analysis with **DABNA-1**^6^. Irrespective of the choice of the DFT functional and the use (or not) of the Tamm-Dancoff approximation, all our TD-DFT calculations yield erroneously large Δ*E*_ST_ values (e.g., 0.56 eV at B3LYP level), in line with refs. ^6,15,16^ (see Supplementary Table 1). The use of tuned range-separated functionals does not help either. By varying the amount of Hartree-Fock-like exchange in the functional, we observe a smooth decrease of Δ*E*_ST_ that reaches a more reasonable 0.25 eV with pure semilocal models (e.g., LDA), but at the cost of an unphysical delocalization of the electronic density over the outer phenyl rings (Supplementary Fig. 1). The discrepancy between experiment and theory has more fundamental grounds, as we discovered by running higher-level Spin-Component Scaling second-order approximate Coupled-Cluster (SCS-CC2) calculations with the def2-TZVP basis set (see SI for further details on these calculations).

In contrast to TD-DFT, SCS-CC2 calculations on **DABNA-1** and compound **2** provide (vertical, i.e., based on ground-state geometry) Δ*E*_ST_ values in excellent agreement with experiments, Table 1. Despite relatively small Δ*E*_ST_ values (<0.2 eV), the lowest singlet electronic excitation in these molecules is strongly dipole-allowed with a *f*_osc_ of 0.31 in **DABNA-1** and (summing over the two degenerated singlet excited states arising from the C_3V_-symmetry) 0.26 in **2**. Most interestingly, similar Δ*E*_ST_ values were obtained for vertical and adiabatic (fully relaxed) excitations, owing to modest nuclear reorganization energies in the S_1_ state (0.12 eV and 0.10 eV for **DABNA-1** and molecule **2**, respectively, Table 1), which should ensure narrow singlet emission as an added virtue.

**Table 1:** Excited state properties

Molecule	*n*	S_0_->S_n_	S_0_->T_n_	∆*E*_ST_ (vert)	∆*E*_ST_ (adiab)	∆*E*_ST_ (exp)	*f*_osc_	*λ*_reorg_
**DABNA-1**	1	3.25	3.10	0.15	0.15	0.15^6^	0.31	0.12
	2	4.23	3.75					
	3	4.28	3.87					
**2**	1*	3.67	3.50	0.17	0.20	0.21^7^	0.13	0.10
	2	4.15	3.74					

Vertical excitation energies for the three lowest-lying triplet and singlet excited states, singlet-triplet energy differences (Δ*E*_ST_) for the vertical and adiabatic transitions, oscillator strengths (*f*_osc_), and S_0_->S_1_ reorganization energies (*λ*_reorg_) obtained for **DABNA-1** and compound **2** with the SCS-CC2 method

^*^S_1_ and T_1_ are doubly degenerate. All energies are given in eV

**Fig. 2:**
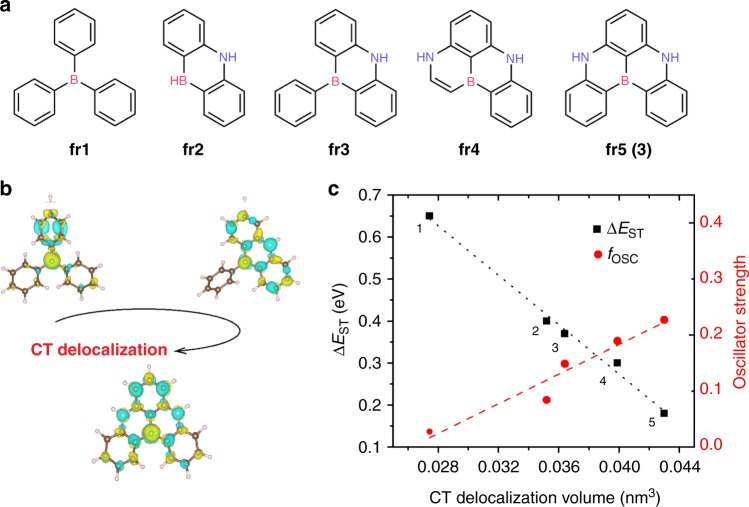
Charge transfer delocalization helps in decreasing the singlet-triplet energy gap and increasing the oscillator strength. **a** Chemical structures of DABNA-1 fragments. **b** Difference density plots computed for selected fragments from **a**. **c** Δ*E*_ST_ (black squares) and oscillator strengths, *f*_osc_ (red dots) as a function of CT delocalization volume for the selected set of fragments

The origin for the unusual electronic properties of these molecules is best pictured by difference density plots for the S_0_-S_1_ and S_0_-T_1_ excitations, calculated at the SCS-CC2 level and shown in Fig. 1b for **DABNA-1** (cf. Supplementary Fig. 1). These plots reveal a remarkably homogenous short-distance reshuffling of the electronic density upon excitation that yields spatially alternating hole-rich and electron-rich regions. The density reorganization is partly shaped by second-order electronic correlation effects (Supplementary Fig. 2) and thus, not surprisingly, cannot be fully captured at the (one-electron) TD-DFT level. To elucidate further the origin for the small Δ*E*_ST_ in **DABNA-1** and **2**, it is instructive to analyze the results obtained for fragments (labeled **fr1–fr5** in Fig. 2a) extracted from the parent molecules. Close inspection of the differential density plots for both singlet and triplet excitations show the same alternating pattern as the one observed for **DABNA-1**, yet with an increasing spreading in space as the fragment becomes larger and/or more conjugated, Fig. 2b. This effect can be quantitatively assessed by computing the CT delocalization volume (see Supplementary Information "Difference density plots" section for computational details). Figure 2c shows strong linear correlations (*R*^2^ = 0.99) between both Δ*E*_ST_ or *f*_osc_ and the CT volume, with more extended systems providing, remarkably, smaller Δ*E*_ST_ and larger *f*_osc_. The trend is in fact reminiscent of the behavior of localized (covalent) excitations in conjugated materials, where the exchange energy measured/calculated in polymer chains is usually significantly smaller than that in the parent small molecules^17^.

### Highly emissive molecules with small Δ*E*_ST_

**Fig. 3:**
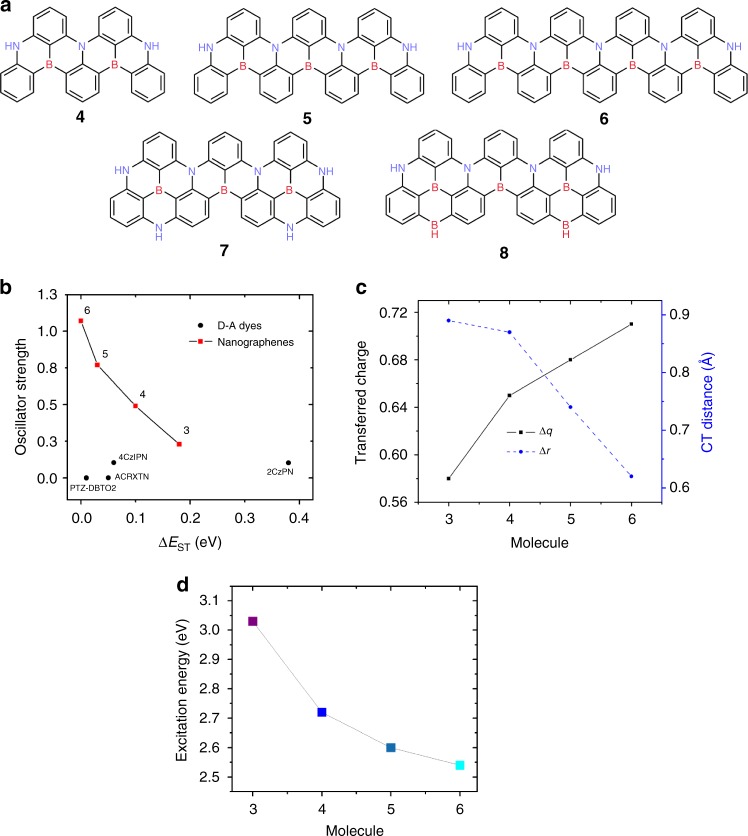
Excited state properties for the designed boron-doped and nitrogen-doped π-extended nanographenes. **a** Chemical structures of engineered molecules **4–8**. Compound **3** coincides with **fr5** in Fig. 2a. **b** Oscillator strength, *f*_osc_, as a function of ∆*E*_ST_ for **3**–**6** (red squares) against conventional D-A reference TADF emitters (black dots, see chemical structures in Supplementary Fig. 3). **c** Transferred charge (in |e|) and CT distance (Å), as computed for molecules **3**–**6**. **d** Emission energies and respective colors for **3**–**6**

The most striking feature arising from Fig. 2c is that while CT delocalization triggers a large decrease in Δ*E*_ST_ from **fr1** (0.65 eV) to **fr5** (0.18 eV), the lowest excitation *f*_osc_ strength instead increases along the same sequence, from 0.02 in **fr1** to 0.23 in **fr5**. This result contrasts with the behavior observed for conventional D-A-based TADF molecular architectures^12^. Inspired by this finding, we applied the multiple charge resonance strategy to design in silico new TADF chromophores based on π-extended B-doped and N-doped nanographenes (Fig. 3a). The results reported in Fig. 3b confirm the trends observed for the fragments in Fig. 2, i.e., reduced Δ*E*_ST_ (as supported by the Zero Field Splitting calculations, see Supplementary Table 4) and enhanced *f*_osc_. In the largest compound investigated, **6**, the singlet and triplet excitations are found to be quasi-degenerate (Δ*E*_ST_ ~3 meV), yet the singlet *f*_osc_ is as large as 1.0. Moreover, as all investigated molecules have a rigid backbone with small (intramolecular) nuclear reorganization energies (Supplementary Table 3), we expect these give rise to narrow linewidth and pure color emission. It is important to stress that the increment in *f*_osc_ is not solely caused by the increase of the number of π-electrons but, instead, occurs primarily through extended delocalization of the wavefunction and increased polarizability along the main-axis of the molecules.

Additional insights into this unexpected behavior can be gained from a detailed analysis of the singlet excited-state wavefunctions, in particular of the following two metrics that assess the relative electron-hole quasi-particles: the electron-hole separation (∆*r*) and the amount of charge transferred (∆*q*). Figure 3c points to a picture where the low-lying singlet excited state undergoes substantial reshuffling of the electronic density (large ∆*q* values) but over short distances (small ∆*r* values). Besides, these characteristics get more pronounced as the ribbons grow longer: ∆*r* decreases from 0.9 to 0.6 Å when going from **3** to **6**, while ∆*q* increases from 0.58 |e| in **3** up to 0.71 |e| in **6**. The resulting ‘short-range/local CT’ states feature both high electron-hole wavefunction overlaps and small exchange interactions, turning into high *f*_osc_ and low Δ*E*_ST_ values for the DABNA core building block. In addition, ‘long-range’ π-conjugation along ribbons **3–6** translates into electronic excitations with extended delocalization and hence increased polarizability and radiative decay rates. While none of these effects is surprising when taken separately, it is the combination of short-range charge separation and long-range electronic delocalization that makes these molecules unique. We believe this is a real breakthrough as most of the low Δ*E*_ST_ TADF molecules reported so far are characterized by very low *f*_osc_ and radiative decay rates. This is exemplified by the comparison of compounds **3–6** with selected D-A TADF reference molecules, for which *f*_osc_ and Δ*E*_ST_ calculated at a similar level of theory (Fig. 3b and Supplementary Table 2) are concurrently small, in line with their ∆*r* values larger than the 1.5–2.0 Å criterion usually set to discriminate CT excitations^18^ that we reported previously^12^.

Finally, besides the beneficial effect on Δ*E*_ST_ and *f*_osc_, increased delocalization along the series **3**–**6** expectedly produces a spectral red-shift of the lowest singlet excitation, thereby allowing for variations of the emission color from blue to green as a function of the molecular size (Fig. 3d). Further tuning of the emission wavelength can be achieved, for instance by introducing an excess amount of N-atoms or B-atoms into the conjugated plane. For the sake of illustration, we designed two other molecules, **7** and **8**, based on **5**. These chromophores essentially retain the properties of the parent molecule in terms of Δ*E*_ST_ and *f*_osc_ (see Supplementary Table 3), however, they possess emission wavelengths blue-shifted to 2.85 eV (435 nm, deep blue, **7**) and red-shifted to 2.1 eV (590 nm, yellow,  **8**) compared to **5**.

To conclude, using highly correlated wavefunction-based methods, we have elucidated the origin for the reduced singlet-triplet gap in the boron-centered azatriangulene molecules originally reported by Hatakeyama et al. as resulting from a local alternating rearrangement of the electronic density upon excitation. We have further shown that this is compatible with high absorption and emission extinction coefficients and we have proposed a strategy, based on promoting short-range charge-transfer in π-extended chromophores, as a way to simultaneously optimize RISC and radiative decay rates. This approach has been successfully applied to the design of TADF molecules with: (i) close-to-resonant lowest singlet and triplet excited states; (ii) large singlet radiative decay rates; (iii) tunable and expected pure color singlet emission. We hope our theoretical work will catalyze the synthesis and trigger the characterization of a new generation of TADF molecules with unprecedented electroluminescence quantum efficiencies.

## Methods

### Computational details

The SCS-CC2 calculations were performed by the TURBOMOLE 6.5 software^19^ using the spin-adapted formulation of the linear response theory and the def2-TZVP basis set^20^. The TD-DFT calculations were carried out using the def2-TZVP basis set. The TD-DFT calculations together with the respective analysis, were performed with the ORCA 4.0.1.2 software^21^.

## Supplementary information

Supplementary Information

## Data Availability

All data are available from the corresponding author upon request.
