# Supplementary material for: Highly emissive excitons with reduced exchange energy in thermally activated delayed fluorescent molecules
Source: Nat Commun. 2019 Feb 5;10:597. doi: 10.1038/s41467-019-08495-5 (PMC6363735; doi:10.1038/s41467-019-08495-5)
Supplement: Supplementary file 1 — Supplementary Information [file 41467_2019_8495_MOESM1_ESM.docx]

**Highly emissive excitons with reduced exchange energy in thermally activated delayed fluorescent molecules**

Anton Pershin,^1^ David Hall,^1,2^ Vincent Lemaur,^1^ Juan-Carlos Sancho-Garcia,^3^ Luca Muccioli,^4^ Eli Zysman-Colman,^2^ David Beljonne,^1^ and Yoann Olivier^1^

^1^Laboratory for Chemistry of Novel Materials, University of Mons, Place du Parc 20, B-7000 Mons, Belgium.

^2^Organic Semiconductor Centre, EaStCHEM School of Chemistry, University of St Andrews, St Andrews, United Kingdom, KY16 9ST.

^3^Departamento de Química Física, Universidad de Alicante, E-03080 Alicante, Spain.

^4^Dipartimento di Chimica Industriale “Toso Montanari”, Università di Bologna, I-40136 Bologna, Italy

# Supplementary notes

# **Comparison between DFT and ab initio methods.**

The majority of results reported in the paper were obtained by using SCS-CC2 method [1]. This second-order perturbation theory approach is very similar to CC2 [2], which is by itself a robust electronic structure method [3], while SCS is an empirical correction scheme [4] based on spin-component scaling and designed specifically to improve the accuracy of excited states description. In fact, as shown thereafter, f_osc_ and ΔE_ST_ values computed by both methods are found to be almost identical for the majority of molecules investigated here, with CC2 excitation energies being systematically ~0.2 eV lower. Moreover, these methods were benchmarked against state-of-the-art Similarity Transformed Equation-Of-Motion Coupled Cluster Singles and Doubles (STEOM-CCSD) calculations for **DABNA-1**, finding very good agreement with the SCS-CC2 results. All these higher-order methods give excellent agreement with the experimental ΔE_ST_ value (0.15 eV [5]). In addition, we also computed the electronic properties of **DABNA-1** by the linear-response TD-DFT approach with several functionals to isolate the effect of the latter on the results, and, in particular, of the fraction of Hartree-Fock-like exchange. The SCS-CC2 calculations, including geometry optimization, were performed by the TURBOMOLE 6.5 software [6] using the spin-adapted formulation of the linear response theory. Unless otherwise indicated, the results were obtained with the def2-TZVP basis set [7]. Note that increasing the size of basis sets from cc-pVDZ to def2-TZVP resulted in a slight red-shift in the calculated S_1_ and T_1_ energies (see Supplementary Table 1) while ΔE_ST_ remained essentially unaffected. The STEOM-CCSD and TD-DFT calculations, together with the respective analysis, were performed with the ORCA 4.0.1.2 software [8]. The reorganization energies were computed at S_1_ potential energy surface (PES) as λ=E(S_1_)//E(GS) - E(S_1_) , where E(S_1_)//E(GS) corresponds to the energy of S_1_ calculated at the ground state geometry and E(S_1_) is the energy of the optimized S_1_ excited state.

Supplementary Table 1: Singlet and triplet excitation energies obtained at various levels of theory for DABNA-1. Energies are provided in units of eV. Here, vertical excitation energies are compared for different electronic structure methods at PBE0 ground state geometry. All transitions are HOMO-> LUMO with the minor admixture of other excitations (none of them exceeding 3 %).

|  |  |  |  |  |
| --- | --- | --- | --- | --- |
| **Method** | **Type** | **S_0_->S_n_** | **S_0_->T_n_** | **ΔE_ST_** |
| VWN | LDA | 2.67 | 2.42 | 0.25 |
| PBE | GGA | 2.65 | 2.30 | 0.34 |
| PBE0 | HYB (25 % HF ex) | 3.19 | 2.67 | 0.53 |
| BH-LYP | HYB (50 % HF ex) | 3.68 | 2.91 | 0.76 |
| ωB97 | ω=0.17 | 3.27 | 2.75 | 0.52 |
| HF | CIS | 4.49 | 3.34 | 1.14 |
| **CC2** | cc-pVDZ | 3.13 | 2.94 | 0.19 |
|  | def2-TZVP | 3.04 | 2.87 | 0.17 |
| **SCS-CC2** | cc-pVDZ | 3.32 | 3.15 | 0.17 |
|  | def2-TZVP | 3.25 | 3.10 | 0.15 |
| **STEOM-CCSD** | cc-pVDZ | 2.98 | 2.86 | 0.12 |
| **Experimental** |  |  |  | **0.15** |

#

# **Difference density plots**

The difference density plots were computed from the (relaxed) real space electron densities of the excited and ground states. In this work, the CT delocalization volume (Figure 2c) is defined as a real physical volume, which belongs to the CT excitation. Inspired by the calculation of the molecular volume from the electron density counter by means of Monte Carlo integration [9] we computed the CT delocalization volume following a similar procedure while based on the absolute values of difference density counter$(\int\left| \Delta\rho\right|dV)$. Further analysis of the difference density plots was carried out in terms of transferred charge and CT distance [10]. The transferred charge was computed by integrating the spatial distributions of the positive part of the difference densities, while CT distance corresponds to the difference between two barycenters of negative and positive densities. These calculations were carried out by Multiwfn code [11].


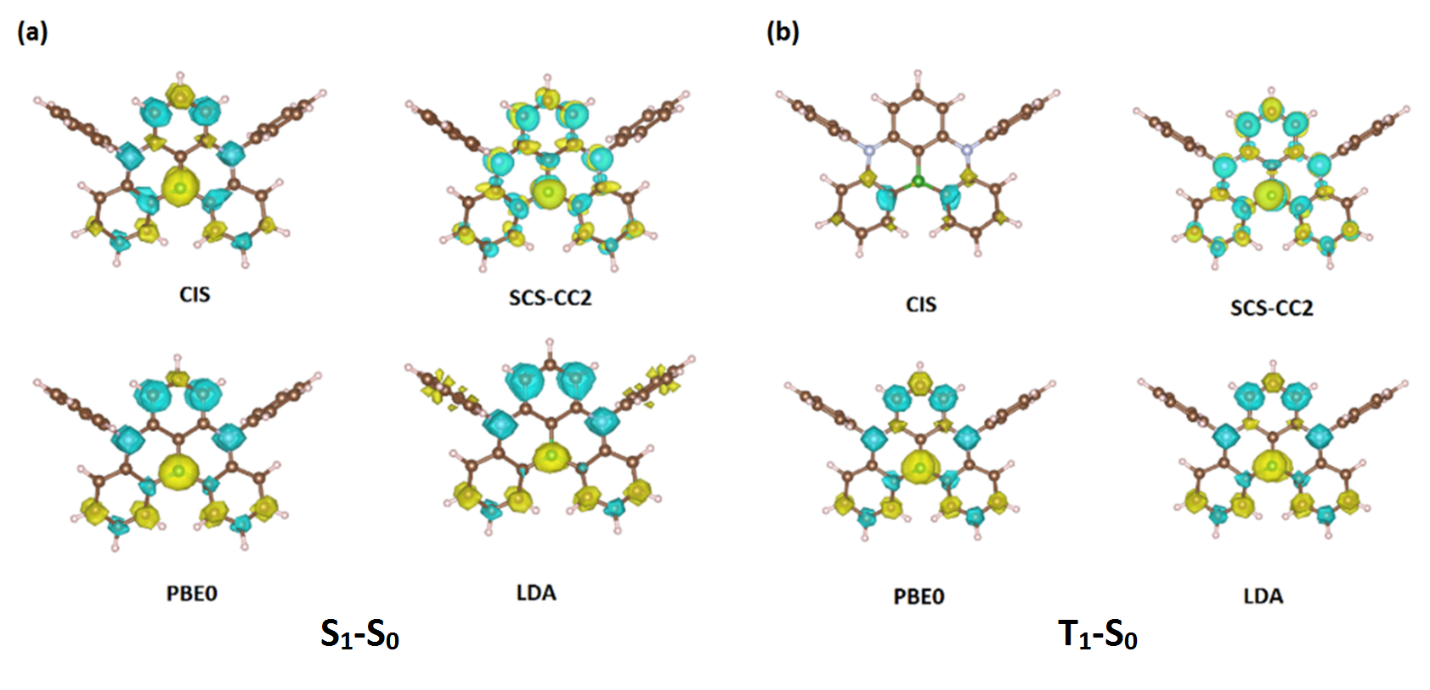


Supplementary Figure 1. Difference density plots for **DABNA-1**, obtained at the selected levels of theory for (a) S_1_-S_0_ and (b) T_1_-S_0_ transitions. Yellow color indicates increased electron density upon excitation, while cyan color is for decreased electron density.

To elucidate further the impact of second-order effects, the corresponding density difference, based on natural transition orbitals, was calculated and plotted in Figure S2. We note that for all compounds the contribution of double excitations amounts to around 10%. However, the impact on the S_1_ energies and thus on the ΔE_ST_ is rather different when considering D-A and boron-centered triangulene-like TADF emitters. For D-A systems, because of the rather small overlap between the hole and electron densities, the correction does not affect too much the predicted ΔE_ST_ gap. Taking **2CzPN** as an example, we obtained previously a value for ΔE_ST_ of 0.36 eV with Time-Dependent DFT (TD-DFT) within the Tamm-Dancoff approximation (TDA) using the PBE0 functional and the def2-TZVP basis set [12], while the SCS-CC2 calculations using the same basis set led to a very similar ΔE_ST_ of 0.35 eV. Moreover, the difference in density calculated for S_1_ is only slightly affected when considering or not double excitations contributions (see Figure S2, bottom). In boron-centered triangulene-like TADF emitters, the overlap between the hole and electron densities is much larger (cf Figure S2 where cyan and yellow clouds are intermixed for **DABNA-1**, while they are separated in the donor and acceptor moieties, respectively, for **2CzPN**). Therefore, the role of correlation effects appears to be critical in determining ΔE_ST_, and TD-DFT or any quantum chemical method including only single excitations will lead to a strong divergence with respect to correlated methods [13]. To summarize, the inclusion of the correlation effects through the SCS-CC2 scheme involves new contributions in the difference density plots as well as slight density reorganization, which may affect the prediction of the singlet-triplet gap in the case of overlapping hole and electron densities.


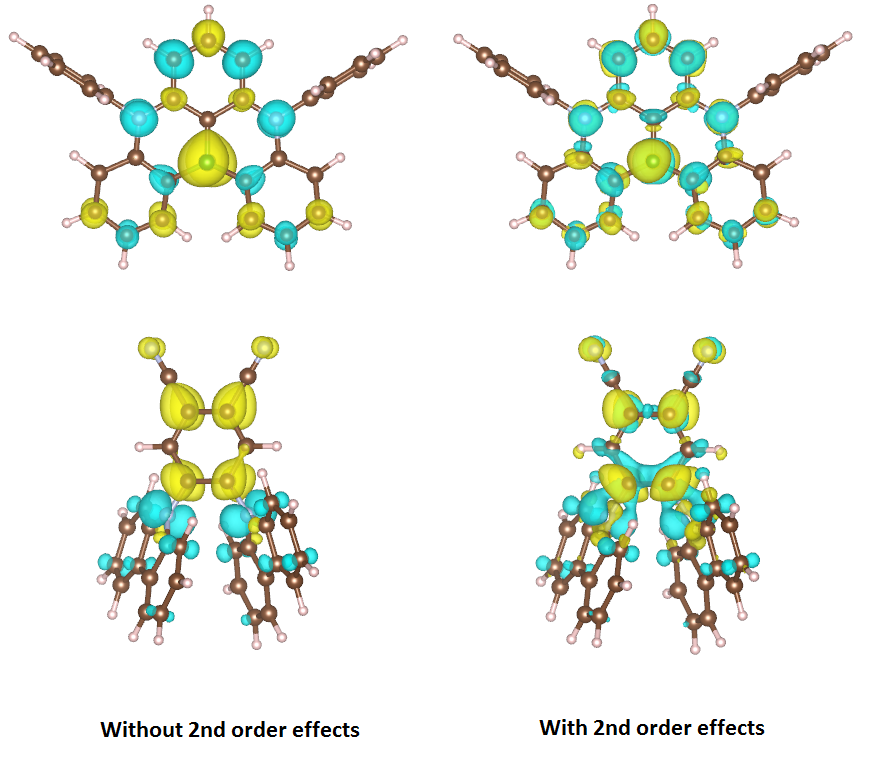


Supplementary Figure 2. Impact of the second order effects (electronic correlation and double excitations) on the charge difference density of S_1_ for **DABNA-1** (top) and **2CzPN** (bottom). Yellow color indicates increased electron density upon excitation, while cyan color is for decreased electron density.

# **Comparison with conventional TADF materials**

We report here the energies of the lower-lying singlet and triplet excited states for a selection of prototypical donor-acceptor TADF materials exhibiting small ΔE_ST_. In comparison to the boron-centered azatriangulene molecules investigated in this study, these compounds display very small oscillator strengths, which translate into low photoluminescence quantum yields. As seen previously, increasing the basis set size from cc-pVDZ to def2-TZVP results in a slight red-shift in the calculated S_1_ and T_1_ energies (see Supplementary Table 2). In contrast, in Supplementary Table 3 we summarize the results for the newly designed nanographenes (see chemical structures in Figure 3).

Supplementary Table 2. Energies (eV) of the first three singlet- and triplet- excitations obtained for the molecules from Figure S3 by SCS-CC2 and CC2 methods.

| **Molecule** | **method** | **basis set** | **S_0_-S_n_** | **S_0_-T_n_** | **method** | **basis set** | **S_0_-S_n_** | **S_0_-T_n_** | **f_osc_** |
| --- | --- | --- | --- | --- | --- | --- | --- | --- | --- |
| 4CzIPN | CC2 | cc-pVDZ | 2.96 | 2.91 | SCS-CC2 | cc-pVDZ | 3.28 | 3.22 | 0.10 |
|  |  |  | 3.24 | 2.99 |  |  | 3.57 | 3.23 |  |
|  |  |  | 3.31 | 3.14 |  |  | 3.67 | 3.45 |  |
|  |  | def2-TZVP | 2.84 | 2.79 |  | def2-TZVP | 3.19 | 3.14 |  |
|  |  |  | 3.12 | 2.92 |  |  | 3.48 | 3.18 |  |
|  |  |  | 3.18 | 3.03 |  |  | 3.57 | 3.38 |  |
| PTZ-DBTO2 | CC2 | cc-pVDZ | 3.44 | 3.41 | SCS-CC2 | cc-pVDZ | 3.90 | 3.58 | 3.2x10^-5^ |
|  |  |  | 3.65 | 3.46 |  |  | 4.00 | 3.59 |  |
|  |  |  | 4.13 | 3.56 |  |  | 4.38 | 4.04 |  |
|  |  | def2-TZVP | 3.30 | 3.29 |  | def2-TZVP | 3.78 | 3.52 |  |
|  |  |  | 3.50 | 3.33 |  |  | 3.87 | 3.54 |  |
|  |  |  | 3.95 | 3.51 |  |  | 4.22 | 3.79 |  |
| ACRXTN | CC2 | cc-pVDZ | 3.33 | 3.30 | SCS-CC2 | cc-pVDZ | 3.77 | 3.68 | 8.5x10^-4^ |
|  |  |  | 3.91 | 3.64 |  |  | 4.14 | 3.85 |  |
|  |  |  | 4.21 | 3.76 |  |  | 4.31 | 3.91 |  |
|  |  | def2-TZVP | 3.13 | 3.10 |  | def2-TZVP | 3.59 | 3.53 |  |
|  |  |  | 3.78 | 3.55 |  |  | 4.03 | 3.79 |  |
|  |  |  | 4.04 | 3.65 |  |  | 4.17 | 3.81 |  |
| 2CzPN | CC2 | cc-pVDZ | 3.44 | 3.17 | SCS-CC2 | cc-pVDZ | 3.74 | 3.35 | 0.12 |
|  |  |  | 3.61 | 3.48 |  |  | 3.89 | 3.74 |  |
|  |  |  | 4.01 | 3.72 |  |  | 4.29 | 3.75 |  |
|  |  | def2-TZVP | 3.33 | 3.09 |  | def2-TZVP | 3.65 | 3.30 |  |
|  |  |  | 3.50 | 3.39 |  |  | 3.80 | 3.68 |  |
|  |  |  | 3.73 | 3.65 |  |  | 4.03 | 3.71 |  |


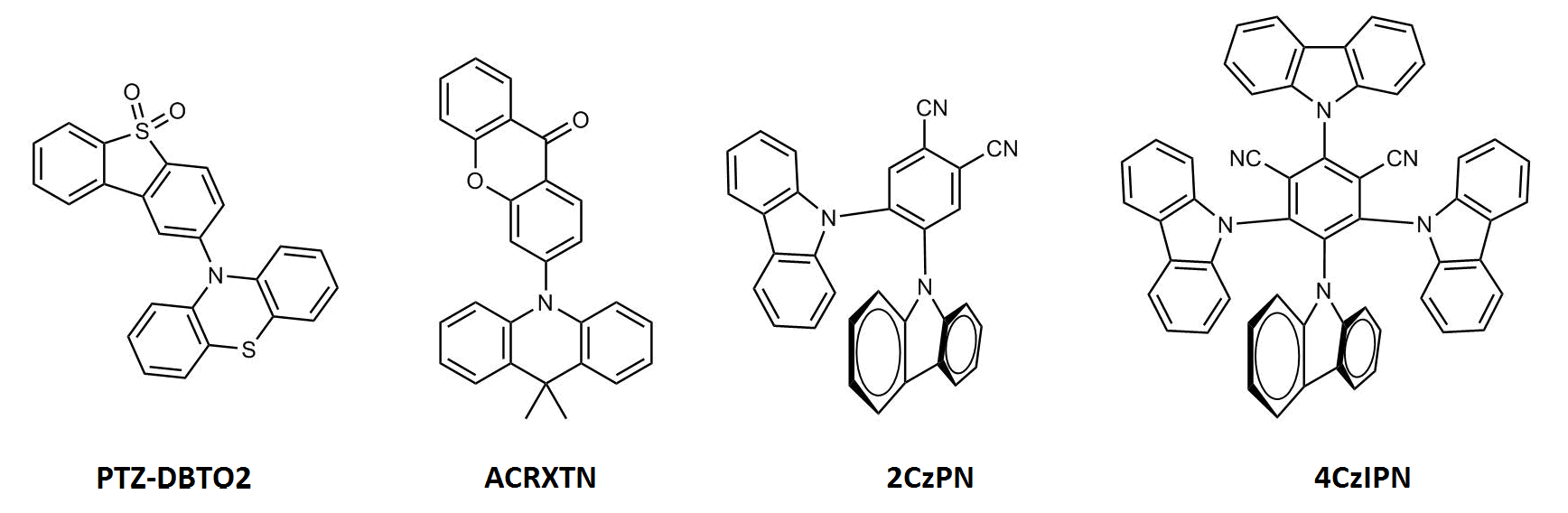


Supplementary Figure 3. Chemical structures of phenothiazine-dibenzothiophene-S,S-dioxide (**PTZ-DBTO2**), 3-(9,9-dimethylacridin-10(9H)-yl)-9H-xanthen-9-one (**ACRXTN**), 4,5-di (9H-carbazol-9-yl) phthalonitrile (**2CzPN**), and 1,2,3,5-tetrakis(carbazol-9-yl)-isophthalonitrile (**4CzIPN**).

Supplementary Table 3. Electronic properties of nanographene molecules from Figure 3a computed by SCS-CC2, including excitation energies (eV), oscillator strengths (as computed, **f_osc_,** and weighted by the number of pi-electrons involved, **f_osc(norm)_,** w.r.t. **3**), reorganization energies (eV), and radiative decay constants from Einstein model (in s^-1^). The molecules were considered in geometries relaxed at S_1_ PES.

| **molecule** | **S_0_-S_1_** | **S_0_-T_1_** | **ΔE_ST_** | **f_osc_** | **f_osc(norm)_** | **λ_reor_** | **k_r_×10^7^** |
| --- | --- | --- | --- | --- | --- | --- | --- |
| 3 | 3.27 | 3.10 | 0.17 | 0.23 | 0.23 | 0.24 | 9.2 |
| 4 | 2.88 | 2.77 | 0.11 | 0.49 | 0.30 | 0.19 | 16.3 |
| 5 | 2.70 | 2.67 | 0.03 | 0.77 | 0.33 | 0.11 | 22.4 |
| 6 | 2.66 | 2.66 | 3x10^-3^ | 1.07 | 0.37 | 0.08 | 29.4 |
| 7 | 2.85 | 2.80 | 0.05 | 0.76 | 0.32 | 0.12 | 24.9 |
| 8 | 2.10 | 2.06 | 0.04 | 0.66 | 0.28 | 0.10 | 16.2 |

# **Zero Field Splitting calculations**

The axial Zero Field Splitting (ZFS) parameter D is related to the interspin distance *r* as [14]:

$$D=\frac{3}{16}\frac{\mu_{0}}{h}\left( g_{e}\mu_{B} \right)^{2}\left\langle\frac{1-3{cos}^{2}\theta}{r^{3}} \right\rangle$$

With θ the angle between the spin-spin vector and the dipolar Z components defined as the polarization axis. D appears as measure of the degree of charge transfer (CT) character in the triplet excited state. The larger the interspin distance is, the larger the CT character in the triplet excited state is, and the lower the D value. As stated in the main manuscript, it is expected that ΔE_ST_ would be reduced when increasing the CT character in the triplet excited state.

We have computed the ZFS parameter D for **DABNA-1** and the designed π-extended B- and N-doped nanographenes at the DFT level (UKS) using the PBE0 functional and the EPR-II basis set [15] optimized for the computation of hyperfine coupling constants with DFT. We have observed a reduction of D (see Supplementary Table 4) when going from compound **3** to **6**, that comes along with a decrease of ΔE_ST_, confirming the increase in the CT character along the series of the designed compounds.

Supplementary Table 4: ZFS parameter D calculated at the PBE0/EPR-II level of theory for DABNA-1 and the designed π-extended B- and N-doped nanographenes.

|  | **3** | **4** | **5** | **6** |
| --- | --- | --- | --- | --- |
| **D (cm^-1^)** | 0.031 | 0.021 | 0.019 | 0.018 |

# **Difference in density plot for compound 2**

Supplementary Figure 4 presents the spatial distribution of the difference of density for the two degenerate states S_1_ and S_2_ for molecule **2** of the main manuscript.


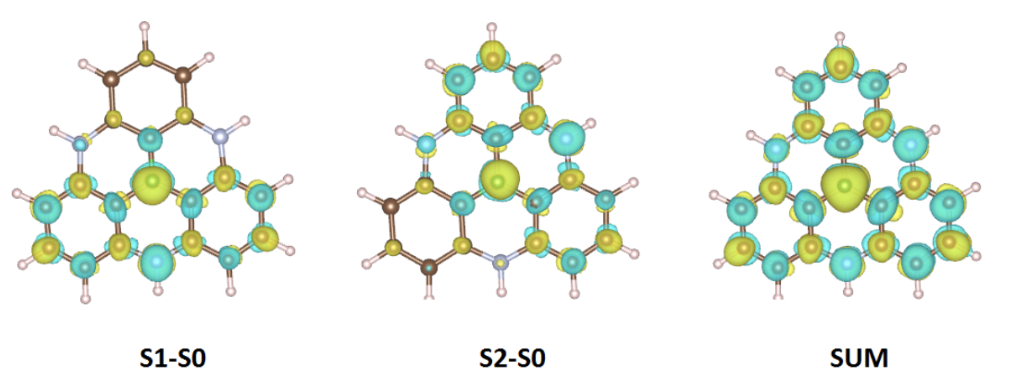


Supplementary Figure 4. Spatial distribution of difference density for the case of two degenerate states S_1_ and S_2_, caused by the presence of C_3_-screw axis.

# **Resonance structures for DABNA-1**

In Supplementary Figure 5, we have presented the most representative resonance structures of DABNA-1.


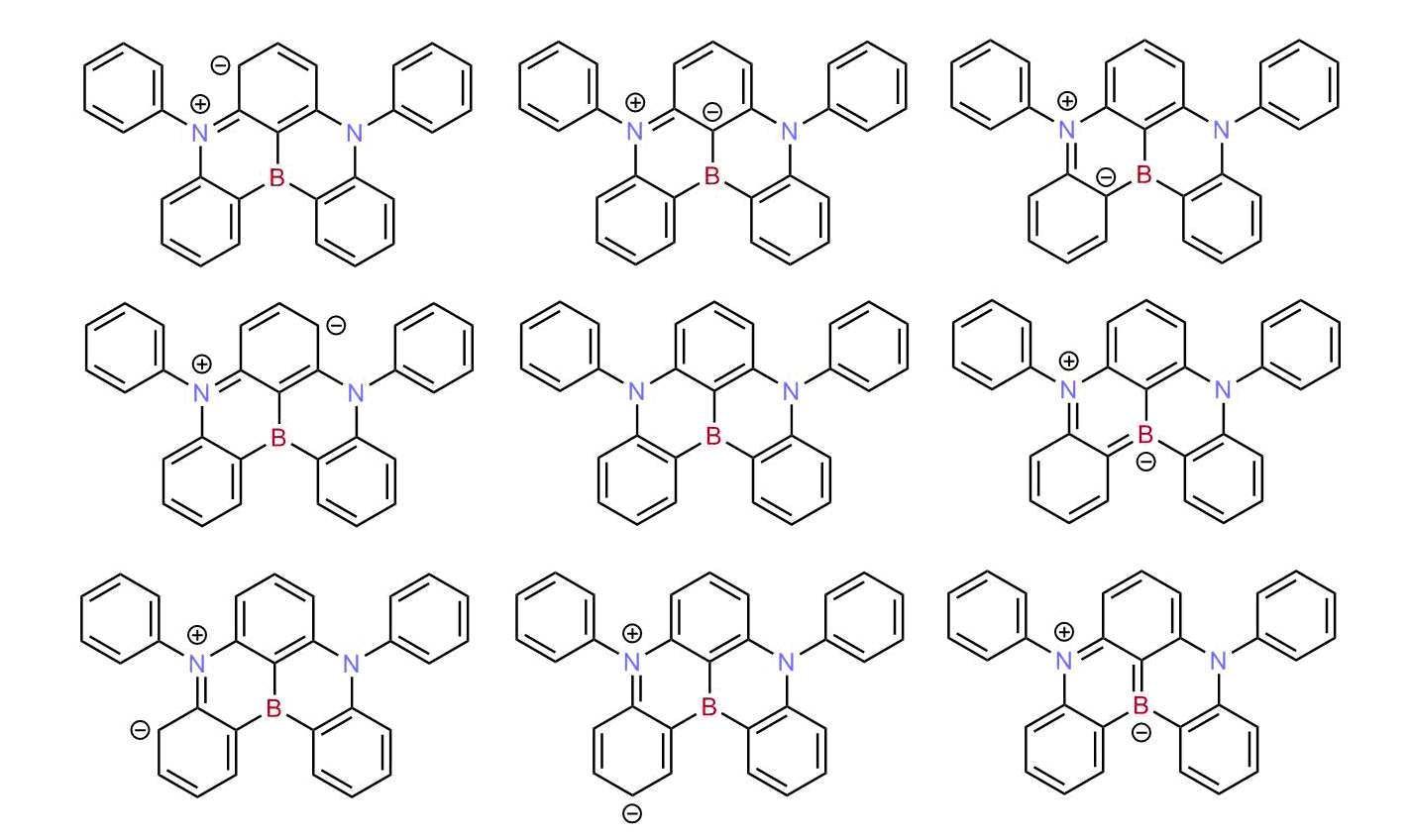


Supplementary Figure 5. Resonance structures in DABNA-1 molecule.

# Supplementary References

| [1] | A. Hellweg, S. A. Grün and C. Hättig. Benchmarking the performance of spin-component scaled CC2 in ground and electronically excited states. *Phys. Chem. Chem. Phys.* **10** (28), 4119-4127 (2008). |
| --- | --- |
| [2] | C. Hättig and F. Weigend. CC2 excitation energy calculations on large molecules using the resolution of the identity approximation. *J. Chem. Phys.* **113** (13), 5154-5161 (2000). |
| [3] | D. Kánnár and P. G. Szalay. Benchmarking coupled cluster methods on valence singlet excited states. *J. Chem. Theory Comput.* **10** (9), 3757-3765 (2014). |
| [4]  [5] | S. Grimme. Improved second-order Møller-Plesset perturbation theory by separate scaling of parallel-and antiparallel-spin pair correlation energies. *J. Chem. Phys.* **118** (20), 9095-9102 (2003).  T. Hatakeyama, K. Shiren, K. Nakajima, S. Nomura, S. Nakatsuka, K. Kinoshita, J. Ni, Y. Ono and T. Ikuta. Ultrapure Blue Thermally Activated Delayed Fluorescence Molecules: Efficient HOMO-LUMO Separation by the Multiple Resonance Effect. *Adv. Mater.* **28** (14), 2777-2781 (2016). |
| [6] | "TURBOMOLE V6.5, a development of University of Karlsruhe and Forschungszentrum Karlsruhe GmbH, 1989-2007, TURBOMOLE GmbH, since 2007; available from http://www.turbomole.com.". |
| [7] | F. Weigend. Error-Balanced Segmented Contracted Gaussian Basis Sets: A Concept and Its Extension to the Lanthanides. *Computational Methods in Lanthanide and Actinide Chemistry.* p. 181, (2015). |
| [8] | F. Neese. Software update: the ORCA program system, version 4.0. *Wiley Interdisciplinary Reviews: Computational Molecular Science.* **8** (1) (2018). |
| [9]  [10]  [11] | M. W. Wong, K. B. Wiberg and M. J. Frisch. Ab initio calculation of molar volumes: comparison with experiment and use in solvation models. *J. Comput. Chem.* **16** (3), 385-394 (1995).  T. Le Bahers, C. Adamo. and I. Ciofini. A qualitative index of spatial extent in charge-transfer excitations. *J. Chem. Theory Comput.* **7** (8), 2498-2506 (2011).  T. Lu and F. Chen. Multiwfn: a multifunctional wavefunction analyzer. *J. Comput. Chem.* **33** (5), 580-592 (2012). |
| [12] | M. Moral, L. Muccioli, W.-J. Son, Y. Olivier and J.-C. Sancho-Garcia. Theoretical rationalization of the singlet-triplet gap in OLEDs materials: impact of charge-transfer character. *J. Chem. Theory Comput.* **11** (1), 168-177 (2014). |
| [13] | S. A. Mewes, F. Plasser, A. Krylov and A. Dreuw. Benchmarking excited-state calculations using exciton properties. *J. Chem. Theory Comput.* **14** (2), 710-725 *(*2018). |
| [14] | S. Richert, C. E. Tait and C. R. Timmel, Delocalisation of photoexcited triplet states probed by transient EPR and hyperfine spectroscopy. *J. Magn. Reson.* **280**, 103-116 (2017). |
| [15] | V. Barone. Structure, magnetic properties and reactivities of open-shell species from density functional and self-consistent hybrid methods. *Recent Advances In Density Functional Methods: (Part I)*, World Scientific, 287-334 (1995). |
